# Associations of specific dietary unsaturated fatty acids with risk of overweight/obesity: population-based cohort study

**DOI:** 10.3389/fnut.2023.1150709

**Published:** 2023-06-08

**Authors:** Weiming Chen, Yang Ao, Xiaochun Lan, Wenzhou Tong, Xiaohui Liu, Xia Zhang, Qiang Ye, Yin Li, Linfen Liu, Hao Ye, Pan Zhuang, Yu Zhang, Weifang Zheng, Jingjing Jiao

**Affiliations:** ^1^Lanxi Red Cross Hospital, Jinhua, Zhejiang, China; ^2^Department of Nutrition, School of Public Health, Zhejiang University School of Medicine, Hangzhou, Zhejiang, China; ^3^Lanxi People’s Hospital, Jinhua, Zhejiang, China; ^4^Lanxi Center for Disease Control and Prevention, Jinhua, China; ^5^Department of Food Science and Nutrition, Fuli Institute of Food Science, College of Biosystems Engineering and Food Science, Zhejiang University, Hangzhou, Zhejiang, China; ^6^Lanxi Hospital of Traditional Chinese Medicine, Jinhua, Zhejiang, China

**Keywords:** unsaturated fatty acids, obesity, overweight, prospective study, China health and nutrition survey

## Abstract

**Background:**

The role of specific unsaturated fatty acids (FAs) in the development of overweight/obesity remains unclear in the general population. Here, we aimed to explore the associations of different types of unsaturated FAs with overweight/obesity risk among the Chinese population.

**Methods:**

Eight thousand seven hundred forty-two subjects free of overweight/obesity at entry in the China Health and Nutrition Survey (CHNS) were followed up until 2015. Dietary unsaturated FAs were assessed by 3-day 24-h recalls with a weighing method in each wave. Cox regression models were used to obtain the hazard ratios (HRs) and 95% confidence intervals (CIs) for overweight/obesity risk associated with unsaturated FAs.

**Results:**

During a median follow-up of 7 years, 2,753 subjects (1,350 males and 1,403 females) developed overweight/obesity. Consuming more monounsaturated FAs (MUFAs) was associated with a lower risk of overweight/obesity (highest vs. lowest quartile: HR 0.80, 95% CI 0.67–0.96; *P*-trend = 0.010). Similar inverse associations were observed for plant-MUFAs (HR_Q4vsQ1_ 0.83, 95% CI: 0.73–0.94; *P*-trend = 0.003) and animal-MUFAs (HR_Q4vsQ1_ 0.77, 95% CI: 0.64–0.94; *P*-trend = 0.004), total dietary oleic acid (OA) (HR_Q4vsQ1_ 0.66, 95% CI: 0.55–0.79; *P*-trend <0.001), plant-OA (HR_Q4vsQ1_ 0.73, 95% CI: 0.64–0.83; *P*-trend <0.001) and animal-OA (HR_Q4vsQ1_ 0.68, 95% CI: 0.55–0.84; *P*-trend <0.001). In addition, the intakes of n-3 polyunsaturated FAs (PUFAs) (HR_Q4vsQ1_ 1.24, 95% CI: 1.09–1.42; *P*-trend = 0.017) and α-linolenic acid (ALA) (HR_Q4vsQ1_ 1.22, 95% CI: 1.07–1.39; *P*-trend = 0.039) but not marine n-3 PUFAs were positively linked to overweight/obesity risk. Consumption of n-6 PUFAs (HR_Q4vsQ1_ 1.13, 95% CI: 0.99–1.28; *P*-trend = 0.014) and linoleic acid (LA) (HR_Q4vsQ1_ 1.11, 95% CI: 0.98–1.26; *P*-trend = 0.020) had marginal and positive relationships with the incidence of overweight/obesity. N-6/n-3 PUFA ratio ranging from 5.7 to 12.6 was related to higher risk of overweight/obesity.

**Conclusion:**

Higher dietary intake of MUFAs was associated with lower overweight/obesity risk, which was mainly driven by dietary OA from either plant or animal sources. Intakes of ALA, n-6 PUFAs and LA were related to higher risk of overweight/obesity. These results support consuming more MUFAs for maintaining a healthy body weight among the Chinese population.

## Introduction

Overweight and obesity are a great burden to the world, with the events of overweight and obesity tripling to 1.6 billion in 2016 compared to 1975s (1). Notably, overweight and obesity are vital risk factors for noncommunicable diseases including cardiovascular disease and cancer, which are leading causes of death (2, 3). China is also threatened by the epidemic of overweight and obesity. The latest data from the Chinese Residents Chronic Disease and Nutrition Surveillance (2020) highlighted that the prevalence of overweight and obesity has rapidly increased to approximately over 50% among Chinese adults (4).

Dietary habits are key modifiable factors to prevent a large fraction of overweight and obesity (5). Among them, different types of dietary fatty acids (FAs) have received great attention in human health (6). Dietary guidelines recommend reducing saturated FA (SFA) intake while increasing the intake of polyunsaturated FAs (PUFAs) and monounsaturated FAs (MUFAs) (7). However, these guidelines are based on cardiovascular benefits and fail to focus on specific FAs that could have divergent effects on body size. Previous studies summarized that the chain length, degree of unsaturation, and position and stereoisomeric configuration of the double bonds of FAs might affect FA oxidation rate thereby influencing body weight (8). Our previous study assessed the association of different chain-length SFA intake with overweight/obesity in the Chinese population, which indicated heterogeneous effects among SFAs with different chain lengths (9). In terms of unsaturated FAs, previous studies suggested that dietary oleic acid (OA) and long-chain n-3 PUFAs had a protective effect on body weight or composition (10, 11), while dietary intake of n-6 PUFAs including linoleic acid (LA) and arachidonic acid (AA) were supported to promote weight gain (12). Earlier studies have revealed different effects of MUFAs from animal and plant sources on human health (13–15). However, prospective studies assessing the effects on the development of overweight/obesity are lacking. Chinese adults have higher consumption of OA and LA, but lower intake of palmitoleic acid (PA), α-linolenic acid (ALA) and marine n-3 PUFAs including eicosapentaenoic acid (EPA) and docosahexaenoic acid (DHA) (16). The current level of unsaturated FA consumption in relation to overweight/obesity development has not been assessed at a nationwide level in China.

To provide further evidence and advance the field, we investigated the diverse associations of different unsaturated FA intakes with the risk of overweight and obesity among 8,742 Chinese adults enrolled in the China Health and Nutrition Survey (CHNS).

## Methods

### Study population

CHNS is an ongoing cohort using multistage cluster random sampling to draw a sample of 30,000 individuals from 15 provinces and municipal cities in China. The CHNS was initiated in 1989. Subsequently, the surveys were conducted in 1991, 1993, 1997, 2000, 2004, 2006, 2009, 2011, and 2015. Detailed procedures have been described elsewhere (17, 18). Given that only adults aged 20–45 y were included in the 1989 round and the food codes from 1991 to 1993 round did not match the food codes in the Chinese Food Composition Table (FCT), participants in the current analysis were recruited from 1997 to 2011 round. We further excluded the participants aged under 20 years old (*n* = 8,706), without complete dietary data based on a 3-day 24-h dietary recall (*n* = 720), had a history of cardiovascular disease (CVD) or cancer at baseline (*n* = 550), with extreme energy intake (< 800 or > 4,200 kcal/day for men and < 600 or > 3,500 kcal/day for women, *n* = 181), without follow-up or with overweight/obesity at baseline (*n* = 8,005), and without BMI data during the follow-up (*n* = 2,572). Finally, 8,742 participants were selected in the present analyzes (Supplementary Figure S1).

### Dietary assessment and covariates

In the CHNS, dietary assessments consist of a 3-day 24-h dietary recall for individuals and a weighing inventory for household food consumption at the same 3 days. Other details on dietary data collection have been described elsewhere (17). Nutrient intakes from various foods were calculated using FCT (19–21). The 1991 version of FCT was used in 1997 and 2000 to obtain dietary information, and the 2002 and 2004 versions were combined for other surveys. Cumulative mean values were computed for each nutrient to represent long-term consumption and reduce within-individual heterogeneity. In addition, demographic and lifestyle information was collected as well, including age, sex, physical activity, marital status, nationality, education level, household income, smoking, alcohol consumption, and history of hypertension and diabetes.

### Ascertainment of overweight and obesity

The height and weight of each participant in each interview were measured by well-trained staffs with the use of standard protocol and instruments. BMI was calculated as body weight (kg) divided by height squared (m^2^). The ascertainment of overweight and obesity was according to the Chinese Criteria of Weight for Adults (WS/T 428–2013): participants with a range of 24 kg/m^2^ ≤ BMI < 28 kg/m^2^ were considered as overweight, while a BMI ≥ 28 kg/m^2^ was considered as obesity.

### Statistical analyzes

Intakes of individual unsaturated FAs were expressed as percentages of total energy intake and then divided into quartiles (22). The baseline characteristics of participants were expressed as the means ± standard errors for continuous variables, while categorical variables were expressed as the percentages (%). To compare proportions or means of baseline characteristics among quartiles of MUFA or PUFA intake, chi-square test for categorical variables and analysis of variance (ANOVA) for continuous variables were applied. The follow-up duration of each participant was calculated from the baseline year to the year of developing overweight/obesity or the date of their last assessment. Multivariable Cox proportional hazards regression models were conducted to estimate the hazard ratios (HRs) and 95% confidence intervals (CIs) for overweight/obesity risk with the first category of unsaturated FAs as the reference. Tests for trends were assessed by calculating the median values in each quartile as continuous variables. Three stepwise models were established with potential confounders considered as covariates: model 1 was a crude model adjusted for age and gender; model 2 was further adjusted for BMI (in kg/m^2^: < 18.5, 18.5–23.9, 24–27.9 or ≥ 28), nationality (Han or non-Han), education (less than high school, high school, some college or at least college), deprivation index (quartile), marital status (never married, married or living as married, widowed/divorced/separated, or unknown), household income (quartile), physical activity (no regular activity, low to moderate activity, or vigorous activity), smoking (never, former, current, or unknown), alcohol drinking status (non-drinker or drinker), history of hypertension (yes, no, or unknown) and diabetes (yes, no, or unknown); model 3 was additionally adjusted for the intake of total energy, percentages of energy from protein, SFAs and remaining FAs where appropriate. For the possible dose–response relationship between individual FAs and overweight/obesity, restricted cubic spline regression was performed with 4 knots at prespecified locations according to the percentiles of FAs.

Subgroup analyzes were conducted stratified by gender, age, smoking status, alcohol consumption, physical activity, education level, household income, and history of hypertension and diabetes. In sensitivity analysis, to test the robustness of models, we further adjusted for cholesterol intake, occupation and alternative healthy eating index (AHEI) (23), excluded participants with extreme BMI (< 18.5 kg/m^2^), and excluded participants with hypertension or diabetes at baseline.

All these analyzes were performed with the use of SAS version 9.4 (SAS Institute, Cary, NC, United States). Two-sided probability values <0.05 were considered statistically significant.

## Results

### Baseline characteristics

The baseline characteristics of 8,742 participants according to quartiles of total MUFA and PUFA intakes are shown in Table 1. Participants who consumed more MUFA or PUFA were older (*p* value <0.001) and women (*p* value <0.001). Furthermore, they had higher household income (*p* value <0.001), higher education levels (*p* value <0.001), higher intake of SFAs (*p* value <0.001) and cholesterol (*p* value <0.001), and higher prevalence of diabetes (*p* value = 0.005 for MUFA, *p* value <0.001 for PUFA). On the contrary, they smoked (*p* value <0.001) and drank alcohol (*p* value <0.001) less frequently, and had less physical activity (*p* value <0.001) and total energy intake (*p* value <0.001).

**Table 1:** Characteristics of the participants at baseline classified by the quartiles of MUFA and PUFA intakes.

	MUFA intake (percentage of energy, %)					PUFA intake (percentage of energy, %)				
	Q1(≤ 8.7)	Q2(8.7–11.4)	Q3(11.4–14.1)	Q4(≥ 14.1)	*P* value	Q1(≤ 5.2)	Q2(5.2–7.0)	Q3(7.0–9.1)	Q4(≥ 9.1)	*P* value
*N*	2,185	2,186	2,186	2,185		2,185	2,186	2,186	2,185	
Age (years)	41.3 ± 0.3	42.3 ± 0.3	43.3 ± 0.3	44.8 ± 0.3	<0.001	42.0 ± 0.3	42.8 ± 0.3	43.2 ± 0.3	43.8 ± 0.3	<0.001
Body mass index (kg/m^2^)	21.0 ± 0.04	21.0 ± 0.04	21.0 ± 0.04	21.0 ± 0.04	0.250	20.8 ± 0.04	20.9 ± 0.04	21.0 ± 0.04	21.2 ± 0.04	<0.001
Household income (yuan/yr)	19,353.2 ± 556.1	27,425.5 ± 697.7	31,293.9 ± 806.8	34,929.0 ± 888.9	<0.001	24,297.2 ± 647.7	29,075.0 ± 751.3	29,129.1 ± 814.2	30,472.7 ± 800.0	<0.001
Male (%)	52.5	47.9	45.8	41.4	<0.001	54.2	49.1	45.0	39.2	<0.001
Han (%)	85.7	84.9	87.4	89.2	<0.001	74.8	86.1	91.7	94.5	<0.001
Married (%)	84.0	85.9	84.2	82.0	<0.001	84.3	84.5	83.8	83.6	<0.001
Greater than high school (%)	5.4	12.5	15.6	17.6	<0.001	7.3	12.5	13.9	17.4	<0.001
Moderate-to-vigorous activity (%)	70.8	57.8	44.4	37.0	<0.001	68.3	54.0	48.4	38.3	<0.001
Current drinker (%)	37.3	37.5	32.8	31.4	<0.001	37.9	36.0	33.9	31.1	<0.001
Current smoker (%)	37.8	31.5	28.7	26.5	<0.001	37.2	31.9	29.6	25.8	<0.001
History of hypertension (%)	9.8	10.2	10.9	10.3	0.712	10.3	10.3	10.8	9.8	0.701
History of diabetes (%)	1.1	1.9	2.5	3.2	0.005	0.9	2.4	2.0	2.5	<0.001
Dietary intake										
Total energy (kcal/day)	2,130.1 ± 11.3	2,108.4 ± 10.5	2,077.6 ± 10.2	2,060.2 ± 11.2	<0.001	2,111.0 ± 11.1	2,128.4 ± 11.0	2,083.5 ± 10.7	2,053.4 ± 10.5	<0.001
Protein (%)	12.1 ± 0.1	12.5 ± 0.1	12.8 ± 0.1	13.1 ± 0.1	<0.001	12.3 ± 0.1	12.8 ± 0.1	12.8 ± 0.1	12.6 ± 0.1	<0.001
Saturated fatty acids (%)	4.7 ± 0.03	6.9 ± 0.03	8.4 ± 0.03	11.3 ± 0.2	<0.001	7.1 ± 0.07	7.7 ± 0.07	7.6 ± 0.06	8.9 ± 0.24	<0.001
Cholesterol (mg/day)	198.8 ± 3.9	285.2 ± 4.9	339.2 ± 4.6	391.2 ± 4.8	<0.001	231.0 ± 4.4	296.5 ± 4.4	328.8 ± 4.9	358.0 ± 5.0	<0.001
AHEI score	53.8 ± 0.2	51.1 ± 0.2	49.7 ± 0.2	48.2 ± 0.2	<0.001	46.8 ± 0.2	48.8 ± 0.2	52.2 ± 0.2	55.0 ± 0.2	<0.001

Values are means ± SE or percentages unless stated otherwise. Q, quartiles; MUFA, monounsaturated fatty acid; PUFA, polyunsaturated fatty acid; AHEI, Alternative healthy eating index.

### MUFA intake and risk of overweight and obesity

**Figure 1:**
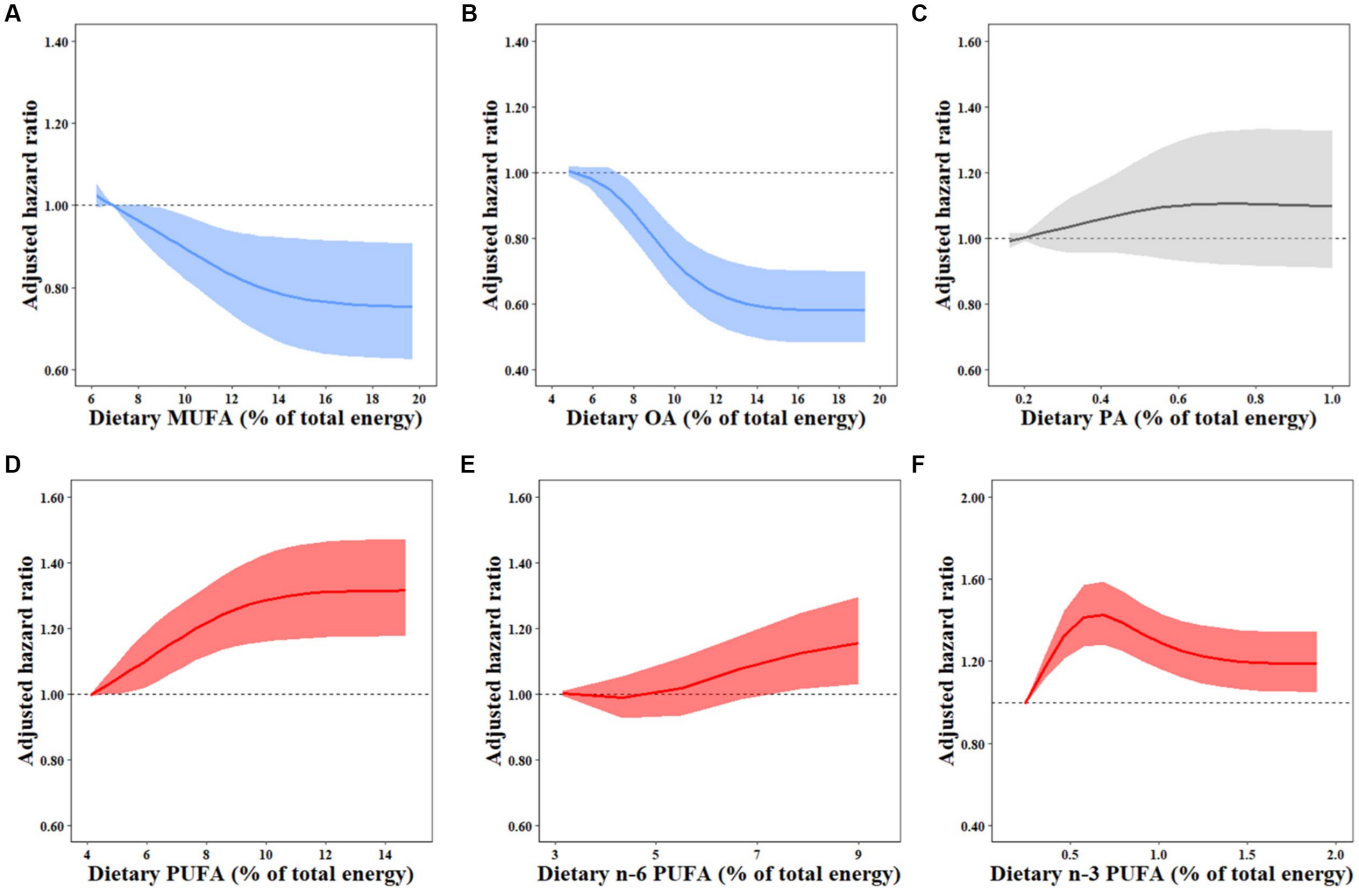
Dose–response relationships between dietary FAs and overweight/obesity risk. HRs for the overweight/obesity risk associated with dietary MUFAs **(A)**, OA **(B)**, PA **(C)**, PUFAs **(D)**, N-6 PUFAs **(E)**, and N-3 PUFAs **(F)** were estimated by restricted cubic-spline regression adjusted for age and sex, marital status, BMI, household income, urbanization index, nationality, education, physical activity, smoking, alcohol drinking status, history of hypertension and diabetes, total energy intake, percentages of energy intake from protein, SFAs, and remaining fatty acids where appropriate. MUFA, monounsaturated fatty acid; PUFA, polyunsaturated fatty acid; OA, oleic acid; PA, palmitoleic acid.

Over a median of 7-year follow-up, 1,350 males and 1,403 females developed overweight/obesity. In model 3 with potential confounders fully adjusted, participants in the highest quartile of MUFA intake presented a significant reduction of 20% in the risk of overweight/obesity (HR_Q4vsQ1_ 0.80, 95% CI: 0.67–0.96; *P*-trend = 0.010; Table 2). The inverse association was also observed for OA intake (HR_Q4vsQ1_ 0.66, 95% CI: 0.55–0.79; *P*-trend <0.001), whereas PA intake was not associated with the risk of overweight/obesity (HR_Q4vsQ1_ 1.15, 95% CI: 0.96–1.37; *P*-trend = 0.132; Table 2). In terms of dietary source of MUFAs, both animal-derived (HR_Q4vsQ1_ 0.77, 95% CI: 0.64–0.94; *P*-trend = 0.004) and plant-derived MUFAs (HR_Q4vsQ1_ 0.83, 95% CI: 0.73–0.94; *P*-trend = 0.003) had inverse associations with overweight/obesity development (Table 3). The results of OA derived from animal (HR_Q4vsQ1_ 0.68, 95% CI: 0.55–0.84; *P*-trend <0.001) and plant sources (HR_Q4vsQ1_ 0.73, 95% CI: 0.64–0.83; *P*-trend <0.001) exhibited similar association patterns (Supplementary Table S1). However, the adjusted HRs and 95% CIs suggested a detrimental association for plant-PA (HR_Q4vsQ1_ 1.29, 95% CI: 1.14–1.47; *P*-trend = 0.002) but not animal-PA consumption (HR_Q4vsQ1_ 0.86, 95% CI: 0.71–1.05; *P*-trend = 0.132; Supplementary Table S1). Restricted cubic spline regression produced similar findings for these MUFAs (Figure 1; Supplementary Figure S2).

**Table 2:** HRs (95% CIs) for the overweight/obesity risk according to MUFA and PUFA consumption.

	Quartiles of dietary fatty acids (% of total energy)				*P-*trend^*^
	Q1	Q2	Q3	Q4	
MUFAs					
Range	≤ 8.7	8.7–11.4	11.4–14.1	≥ 14.1	
Median	6.9	10.2	12.7	16.1	
Cases/person-years	728/19,665	723/15,302	695/15,302	607/13,110	
Model					
1. Overweight/obesity ~ Age, sex	1.00 (ref.)	1.05 (0.94–1.16)	1.05 (0.95–1.17)	1.02 (0.92–1.14)	0.656
2. Overweight/obesity ~ Age, sex, BMI, marital status, household income, urbanization index, nationality, education, physical activity, smoking, alcohol drinking status, history of hypertension and diabetes	1.00 (ref.)	0.97 (0.87–1.08)	0.92 (0.82–1.03)	0.87 (0.77–0.98)	0.016
3. Overweight/obesity ~ Age, sex, BMI, marital status, household income, urbanization index, nationality, education, physical activity, smoking, alcohol drinking status, history of hypertension and diabetes, total energy intake, percentages of energy intake from protein, SFAs, PUFAs	1.00 (ref.)	0.95 (0.84–1.08)	0.86 (0.73–1.00)	0.80 (0.67–0.96)	0.01
OA					
Range	≤ 6.6	6.6–9.0	9.0–11.6	≥ 11.6	
Median	5.1	7.8	10.2	13.8	
Cases/person-years	713/15,295	730/15,302	707/15,302	603/15,295	
Model					
1.Overweight/obesity ~ Age, sex	1.00 (ref.)	1.05 (0.95–1.17)	1.06 (0.96–1.18)	0.95 (0.85–1.06)	0.396
2. Overweight/obesity ~ Age, sex, BMI, marital status, household income, urbanization index, nationality, education, physical activity, smoking, alcohol drinking status, history of hypertension and diabetes	1.00 (ref.)	0.99 (0.89–1.10)	0.95 (0.85–1.07)	0.81 (0.72–0.92)	< 0.001
3. Overweight/obesity ~ Age, sex, BMI, marital status, household income, urbanization index, nationality, education, physical activity, smoking, alcohol drinking status, history of hypertension and diabetes, total energy intake, percentages of energy intake from protein, SFAs, PUFAs, PA	1.00 (ref.)	0.93 (0.82–1.06)	0.83 (0.71–0.96)	0.66 (0.55–0.79)	< 0.001
PA					
Range	≤ 0.3	0.3–0.4	0.4–0.6	≥ 0.6	
Median	0.2	0.4	0.5	0.7	
Cases/person-years	687/19,665	714/19,674	741/15,302	611/13,110	
Model					
1. Overweight/obesity ~ Age, sex	1.00 (ref.)	1.07 (0.97–1.19)	1.21 (1.09–1.34)	1.14 (1.02–1.27)	0.005
2. Overweight/obesity ~ Age, sex, BMI, marital status, household income, urbanization index, nationality, education, physical activity, smoking, alcohol drinking status, history of hypertension and diabetes	1.00 (ref.)	1.03 (0.93–1.15)	1.10 (0.99–1.23)	0.98 (0.86–1.10)	0.755
3. Overweight/obesity ~ Age, sex, BMI, marital status, household income, urbanization index, nationality, education, physical activity, smoking, alcohol drinking status, history of hypertension and diabetes, total energy intake, percentages of energy intake from protein, SFAs, PUFAs, OA	1.00 (ref.)	1.06 (0.93–1.19)	1.16 (1.00–1.35)	1.15 (0.96–1.37)	0.132
PUFAs					
Range	≤ 5.2	5.2–7.0	7.0–9.1	≥ 9.1	
Median	4	6.1	7.9	11.1	
Cases/person-years	641/19,665	659/15,302	734/15,302	719/15,295	
Model					
1. Overweight/obesity ~ Age, sex	1.00 (ref.)	1.09 (0.98–1.21)	1.27 (1.14–1.41)	1.35 (1.22–1.51)	<0.001
2. Overweight/obesity ~ Age, sex, BMI, marital status, household income, urbanization index, nationality, education, physical activity, smoking, alcohol drinking status, history of hypertension and diabetes	1.00 (ref.)	1.03 (0.92–1.15)	1.15 (1.03–1.29)	1.20 (1.07–1.34)	<0.001
3. Overweight/obesity ~ Age, sex, BMI, marital status, household income, urbanization index, nationality, education, physical activity, smoking, alcohol drinking status, history of hypertension and diabetes, total energy intake, percentages of energy intake from protein, SFAs, MUFAs	1.00 (ref.)	1.05 (0.94–1.18)	1.17 (1.04–1.31)	1.24 (1.10–1.39)	<0.001
n-6 PUFAs					
Range	≤ 4.1	4.1–5.6	5.6–7.5	≥ 7.5	
Median	3.1	4.8	6.5	9.6	
Cases/person-years	636/15,295	658/15,302	731/19,674	728/15,295	
Model					
1. Overweight/obesity ~ Age, sex	1.00 (ref.)	1.02 (0.91–1.13)	1.13 (1.01–1.25)	1.28 (1.15–1.43)	<0.001
2. Overweight/obesity ~ Age, sex, BMI, marital status, household income, urbanization index, nationality, education, physical activity, smoking, alcohol drinking status, history of hypertension and diabetes	1.00 (ref.)	0.96 (0.86–1.07)	1.03 (0.92–1.15)	1.14 (1.02–1.28)	0.004
3. Overweight/obesity ~ Age, sex, BMI, marital status, household income, urbanization index, nationality, education, physical activity, smoking, alcohol drinking status, history of hypertension and diabetes, total energy intake, percentages of energy intake from protein, SFAs, MUFAs, n-3 PUFAs	1.00 (ref.)	0.94 (0.84–1.05)	1.02 (0.91–1.14)	1.13 (0.99–1.28)	0.014
n-3 PUFAs					
Range	≤ 0.4	0.4–0.7	0.7–1.1	≥ 1.1	
Median	0.3	0.5	0.9	1.4	
Cases/person-years	590/19,665	705/13,116	737/15,302	721/15,295	
Model					
1. Overweight/obesity ~ Age, sex	1.00 (ref.)	1.49 (1.33–1.66)	1.40 (1.25–1.56)	1.37 (1.22–1.52)	<0.001
2. Overweight/obesity ~ Age, sex, BMI, marital status, household income, urbanization index, nationality, education, physical activity, smoking, alcohol drinking status, history of hypertension and diabetes	1.00 (ref.)	1.42 (1.27–1.58)	1.32 (1.18–1.47)	1.28 (1.14–1.43)	0.003
3. Overweight/obesity ~ Age, sex, BMI, marital status, household income, urbanization index, nationality, education, physical activity, smoking, alcohol drinking status, history of hypertension and diabetes, total energy intake, percentages of energy intake from protein, SFAs, MUFAs, n-6 PUFAs	1.00 (ref.)	1.40 (1.25–1.57)	1.30 (1.16–1.46)	1.24 (1.09–1.42)	0.017
n-6/n-3 PUFA ratio					
Range	≤ 5.7	5.7–7.9	7.9–12.6	≥ 12.6	
Median	4.4	7	9.2	20.5	
Cases/person-years	655/19,665	758/15,302	729/15,302	611/19,665	
Model					
1. Overweight/obesity ~ Age, sex	1.00 (ref.)	1.26 (1.13–1.40)	1.25 (1.12–1.39)	0.93 (0.83–1.04)	<0.001
2. Overweight/obesity ~ Age, sex, BMI, marital status, household income, urbanization index, nationality, education, physical activity, smoking, alcohol drinking status, history of hypertension and diabetes	1.00 (ref.)	1.21 (1.08–1.34)	1.22 (1.09–1.36)	0.93 (0.83–1.04)	0.002
3. Overweight/obesity ~ Age, sex, BMI, marital status, household income, urbanization index, nationality, education, physical activity, smoking, alcohol drinking status, history of hypertension and diabetes, total energy intake, percentages of energy intake from protein, SFAs, MUFAs	1.00 (ref.)	1.20 (1.07–1.35)	1.22 (1.08–1.37)	0.94 (0.84–1.06)	0.003

HRs, hazard risks; CIs, confidence intervals; Q, quartile; MUFA, monounsaturated fatty acid; PUFA, polyunsaturated fatty acid; OA, oleic acid; PA, palmitoleic acid.

* *P*-trend was assessed by calculating the median values in each quartile as continuous variables.

**Table 3:** HRs (95% CIs) for the overweight/obesity risk according to MUFAs from plant and animal sources.

	Quartiles of dietary fatty acids (% of total energy)				*P-*trend^*^
	Q1	Q2	Q3	Q4	
P-MUFAs					
Range	≤ 3.4	3.4–4.9	4.9–7.1	≥ 7.1	
Median	2.5	4.2	6	9.2	
Cases/person-years	610/10,925	712/15,302	754/19,674	677/19,665	
Model					
1.Overweight/obesity ~ Age, sex	1.00 (ref.)	0.95 (0.86–1.06)	0.97 (0.87–1.08)	0.89 (0.80–1.00)	0.056
2. Overweight/obesity ~ Age, sex, BMI, marital status, household income, urbanization index, nationality, education, physical activity, smoking, alcohol drinking status, history of hypertension and diabetes	1.00 (ref.)	0.96 (0.86–1.07)	0.99 (0.89–1.11)	0.90 (0.80–1.01)	0.081
3. Overweight/obesity ~ Age, sex, BMI, marital status, household income, urbanization index, nationality, education, physical activity, smoking, alcohol drinking status, history of hypertension and diabetes, total energy intake, percentages of energy intake from protein, SFAs, PUFAs, A-MUFAs	1.00 (ref.)	0.93 (0.83–1.04)	0.94 (0.84–1.05)	0.83 (0.73–0.94)	0.003
A-MUFAs					
Range	≤ 2.9	2.9–5.0	5.0–7.2	≥ 7.2	
Median	1.5	4	6	9.1	
Cases/person-years	718/19,665	757/19,674	696/15,302	582/13,110	
Model					
1.Overweight/obesity ~ Age, sex	1.00 (ref.)	1.11 (1.00–1.22)	1.05 (0.94–1.16)	1.03 (0.92–1.15)	0.79
2. Overweight/obesity ~ Age, sex, BMI, marital status, household income, urbanization index, nationality, education, physical activity, smoking, alcohol drinking status, history of hypertension and diabetes	1.00 (ref.)	1.01 (0.91–1.13)	0.93 (0.83–1.04)	0.86 (0.76–0.97)	0.005
3. Overweight/obesity ~ Age, sex, BMI, marital status, household income, urbanization index, nationality, education, physical activity, smoking, alcohol drinking status, history of hypertension and diabetes, total energy intake, percentages of energy intake from protein, SFAs, PUFAs, P-MUFAs	1.00 (ref.)	0.99 (0.86–1.12)	0.84 (0.72–0.99)	0.77 (0.64–0.94)	0.004

HRs, hazard risks; CIs, confidence intervals; Q, quartile; P-MUFA, plant-monounsaturated fatty acid; A-MUFA, animal-monounsaturated fatty acid.

* *P*-trend was assessed by calculating the median values in each quartile as continuous variables.

### PUFA intake and risk of overweight and obesity

Total dietary PUFA intake was related to an increased risk of overweight/obesity in fully adjusted model 3 (HR_Q4vsQ1_ 1.24, 95% CI: 1.10–1.39; *P*-trend <0.001; Table 2). Participants in the highest quartiles of n-3 PUFAs (HR_Q4vsQ1_ 1.24, 95% CI: 1.09–1.42; *P*-trend = 0.017) and ALA (HR_Q4vsQ1_ 1.22, 95% CI: 1.07–1.39; *P*_−_trend = 0.039) but not marine n-3 PUFAs (HR_Q4vsQ1_ 0.83, 95% CI: 0.68–1.02; *P*-trend = 0.176) had increased overweight/obesity risk compared to the lowest quartiles (Supplementary Table S2). N-6 PUFA intake was marginally and positively correlated with the risk of overweight/obesity (HR_Q4vsQ1_ 1.13, 95% CI: 0.99–1.28; *P*-trend = 0.014), which was primarily owing to LA (HR _Q4vsQ1_ 1.11, 95% CI: 0.98–1.26; *P*-trend = 0.020) but not AA (HR _Q4vsQ1_ 0.96, 95% CI: 0.82–1.13; *P*-trend = 0.515) intake (Supplementary Table S2). Compared with the lowest quartile, the highest quartile of the n-6/n-3 PUFA ratio was not significantly associated with the incidence of overweight/obesity (HR_Q4vsQ1_ 0.94, 95% CI: 0.84–1.06), but higher risk was observed for the n-6/n-3 PUFA ratio ranging from 5.7 to 12.6 (Table 2). Similar results for these PUFAs were demonstrated by restricted cubic spline regressions (Figure 1; Supplementary Figure S3).

### Subgroup and sensitivity analyzes

Subgroup analyzes showed that the inverse associations of overweight/obesity incidence with total dietary MUFA intake were only significant in women (*P*-interaction = 0.041), non-drinkers (*P*-interaction <0.001), and those with higher education level (*P*-interaction = 0.012) and lower physical activity level (*P*-interaction = 0.040). Moreover, the positive associations of PUFA intake with the risk of overweight/obesity only appeared in participants with lower household income (*P*-interaction = 0.007; Supplementary Table S3). In sensitivity analyzes, the associations between unsaturated FA intake and overweight/obesity incidence were not materially changed after further adjustment for dietary cholesterol intake, occupation and AHEI, excluding participants with extremely lower BMI (BMI < 18.5 kg/m^2^) or those with hypertension or diabetes at baseline (Supplementary Tables S4, S5).

## Discussion

To our knowledge, this prospective study is the first to assess the associations of specific dietary unsaturated FA intake with overweight/obesity development among the Chinese population. After adjustment for major potential risk factors, we found that total MUFA, plant-MUFA, animal-MUFA, plant-OA and animal-OA intake was consistently and inversely associated with the risk of overweight/obesity, while ALA, n-6 PUFA and LA intake was positively related to overweight/obesity risk.

OA is the most common type of MUFAs and is mainly consumed from vegetable oil and pork by the Chinese people (14). The beneficial effect of OA on preventing overweight/obesity was supported by several mechanistic studies. First, an OA-rich diet could increase the fat oxidation rate compared to a high SFA diet (24). In addition, the derivative of OA, oleoylethanolamide (OEA) plays a role in appetite modulation and energy intake (25). Although studies on OA biomarkers came to the contrary conclusion that serum OA concertation was positively linked with incident obesity (26). The difference may be due to the fact that serum OA is not an appropriate biomarker for dietary intake but for *de novo* lipogenesis in humans. The accessible regulator of lipogenic gene expression of endogenously synthesized OA and dietary OA was not similar (27). Due to accumulating epidemiology evidence highlighting the importance of food sources of MUFAs on health (13–15), we further assessed the associations of animal and plant sources of MUFAs/OA with the risk of overweight/obesity and found that both sources in CHNS were consistently associated with a reduced risk of overweight/obesity. Moreover, plant-MUFAs seemed less protective than animal-MUFAs in our study, which could be explained by the common cooking method such as stir-frying and griddling applied to vegetable oils in the daily life of the Chinese. Frying vegetable oil may increase the energy density and trans-FA (TFA) formation (28).

PA is another type of MUFAs and has been evidenced to be beneficial for weight maintenance (29). However, the low intake level of PA consumed in the current analyzes resulted in a null association of total PA or animal-PA with incident overweight/obesity, whereas dietary plant-PA intake was linked to overweight/obesity development. The main source of plant-PA in China was soybean oil (14), which was considered more obesogenic than coconut oil and fructose in mice (30). In addition, the cooking methods of the Chinese may also explain this detected adverse association.

Previous epidemiological studies have confirmed the positive correlation between LA intake and incident overweight/obesity that we identified. A cohort including 20,049 participants with a median of 6.5 years of follow-up concluded that dietary LA intake was positively related to weight gain (31). Similarly, another prospective study conducted in Germany found that the baseline level of erythrocyte LA was associated with a higher overweight/obesity risk in middle-aged and older women during a mean of 10.4-y follow-up (32). Besides, several animal experiments also validated the current findings that dietary n-6 PUFA intake was adipogenic (12, 33). The metabolites of dietary LA, such as anandamide and 2-arachidonyl glycerol, which promoted energy intake and weight gain by reducing hypothalamic satiety signaling and skeletal muscle glucose uptake, and increasing accumulation of lipid droplets in the liver, may be responsible for the effect of LA on body size (12). Moreover, prostacyclin converted from dietary LA could stimulate adipocyte differentiation through several pathways, including activating the peroxisome proliferator-activated receptor (PPAR) family and the CCAAT-enhancer binding protein family (CEBPβ and CEBPδ) (12). For AA, a previous study reported that AA promoted adipogenesis (33), which contradicted the existing conclusion that AA intake was not significantly associated with overweight/obesity development. This discrepancy may mainly be due to the overall low consumption of AA (mean intake: 0.02% kcal/d) in the Chinese population.

ALA is an essential n-3 PUFA and mostly accounted for the positive association of n-3 PUFAs in the current analysis. A cross-sectional study based on the National Health and Nutrition Examination Survey and the What We Eat in America found that the relationship of ALA intake was stratified by some sociodemographic groups as a positive association of ALA with BMI was detected among non-Hispanic black individuals (34). Furthermore, ALA in vegetable oils can be transformed into harmful trans-ALA during stir-frying (35), which may account for the adverse relationship as stir-frying was commonly used for ALA-rich vegetable oils among Chinese people. In addition, ALA-enriched diacylglycerol (ALA-DAG) was more prone to weight gaining compared to ALA-enriched triacylglycerol (ALA-TAG) (36). However, we did not divide ALA into ALA-DAG and ALA-TAG, which may also to some extent explain the harmful association for ALA intake. Although we failed to detect an association between marine n-3 PUFA intake and overweight/obesity risk, the *in vivo* studies have demonstrated that fish oil supplementation, which contained high concentrations of EPA and DHA, could offset weight gain induced by a high-fat diet (11, 37). The mechanism of long-chain n-3 PUFAs could be briefly proposed as stimulating lipid oxidation (38), enhancing satiety (39), and inducing browning of white adipose tissue (40). The extremely low consumption of long-chain n-3 PUFAs in our study has a large gap to the 250 mg/d as international dietary guideline recommends, which probably biased the associations toward the null.

The competition on the same enzyme of LA and ALA during the production of AA and EPA/DHA provided a basic theory of the n-6/n-3 PUFA ratio (41). Previous studies focusing on the ratio of n-6 to n-3 PUFAs summarized a positive association between dietary n-6/n-3 PUFA ratio and overweight/obesity incidence (42), which was generally consistent with our results. However, we found divergent associations between dietary LA and AA, and the difference was also observed between ALA and marine n-3 PUFAs. Our findings indicate that the n-6/n-3 PUFA ratio may not be a proper measurement linking unsaturated FA intake to incident overweight/obesity, whereas specific types of unsaturated FAs should be considered when increasing the intake of unsaturated FAs.

In subgroup analyzes, the association of overweight/obesity with total dietary MUFA intake was only significant in women, non-drinkers, and those with higher education levels and lower physical activity levels. These interactions may due to the higher intake levels of MUFAs among persons with the above characteristics. Other detected interactions remain to be elucidated in future studies.

The current study has some strengths. First, the large population and long-term follow-up could reduce the probability of reverse causation. In addition, cumulative intake of unsaturated FAs was used to represent a long-term diet, and within-individual heterogeneity could be reduced as well. Furthermore, this study systematically assessed the associations of different types of unsaturated FAs from diet with overweight/obesity risk. Despite these strengths, some limitations should also be recognized in the current study. First, measurement bias could not be controlled completely, but using the cumulative average intake of nutrients helped to reduce measurement errors. Second, although we adjusted for many potential confounders in models, unmeasured factors still remained and may influence observed results. Third, dietary TFAs was not adjusted in models due to unavailable data. However, the consumption of dietary TFAs was very low in China (43), which may not significantly change our documented results. Fourth, the findings may not apply to other populations because the cooking style and dietary patterns were unique to the Chinese population. Finally, it was not sufficient to establish causality due to the observational nature of this study.

In conclusion, the current findings supported that total dietary MUFA intake was inversely associated with the risk of overweight/obesity, which was mainly driven by dietary OA from both plant and animal sources. ALA and LA had strong positive associations with overweight/obesity risk. Our findings emphasize the importance of increasing the consumption of MUFAs, especially OA, in overweight/obesity prevention among the Chinese population.

## Data availability statement

Publicly available datasets were analyzed in this study. This data can be found at: https://www.cpc.unc.edu/projects/china/data/. The raw data supporting the conclusions of this article will be made available by the authors, without undue reservation.

## Ethics statement

The studies involving human participants were reviewed and approved by the institutional review boards at the University of North Carolina, Chapel Hill and the National Institute of Nutrition and Food Safety from the Chinese Center for Disease Control and Prevention. The patients/participants provided their written informed consent to participate in this study.

## Author contributions

YZ, WZ, and JJ conceived and designed the study. YA, XHL, and PZ did the data cleaning, analysis and interpretation. YA wrote the manuscript. WC, YA, XCL, YL, HY, PZ, YZ, and JJ were involved in data acquisition. JJ is the guarantor. All authors contributed to the interpretation of the data and critical revision of the manuscript for important intellectual content and approved the final draft.

## Funding

The study was supported by the Zhejiang Provincial Natural Science Foundation of China (Grant Number: LZ20C200001). The funders had no role in design and conduct of the study; collection, management, analysis, and interpretation of the data; preparation, review, and approval of the manuscript; or the decision to submit the manuscript for publication.

## Conflict of interest

The authors declare that the research was conducted in the absence of any commercial or financial relationships that could be construed as a potential conflict of interest.

## Publisher’s note

All claims expressed in this article are solely those of the authors and do not necessarily represent those of their affiliated organizations, or those of the publisher, the editors and the reviewers. Any product that may be evaluated in this article, or claim that may be made by its manufacturer, is not guaranteed or endorsed by the publisher.
