# Supplementary material for: Associations of specific dietary unsaturated fatty acids with risk of overweight/obesity: population-based cohort study
Source: Front Nutr. 2023 Jun 8;10:1150709. doi: 10.3389/fnut.2023.1150709 (PMC10285060; doi:10.3389/fnut.2023.1150709)
Supplement: Supplementary file 1 [file Data_Sheet_1.docx]

# Supplementary Materials

[Supplementary Figure 1. Flow chart of study participants for analyses 2](#_Toc120893016)

[Supplementary Figure 2. Dose-response relationships between dietary MUFA intake and overweight/obesity risk.. 3](#_Toc120893017)

[Supplementary Figure 3. Dose-response relationships between dietary PUFA intake and overweight/obesity risk. 4](#_Toc120893018)

[Supplementary Table 1. Multivariable HRs (95% CIs) of overweight/obesity incidence according to the dietary intake of OA/PA from plants and animals 5](#_Toc120893019)

[Supplementary Table 2. Multivariable HRs (95% CIs) of overweight/obesity incidence according to PUFA subclass 8](#_Toc120893020)

[Supplementary Table 3: Subgroup analyses for multivariable HRs (95% CIs) of overweight/obesity incidence according to specific unsaturated FA intake 11](#_Toc120893021)

[Supplementary Table 4: Sensitivity analyses for multivariable HRs (95% CIs) of overweight/obesity incidence according to dietary MUFA intake 13](#_Toc120893022)

[Supplementary Table 5: Sensitivity analyses for multivariable HRs (95% CIs) of overweight/obesity incidence according to dietary PUFA intake 16](#_Toc120893023)


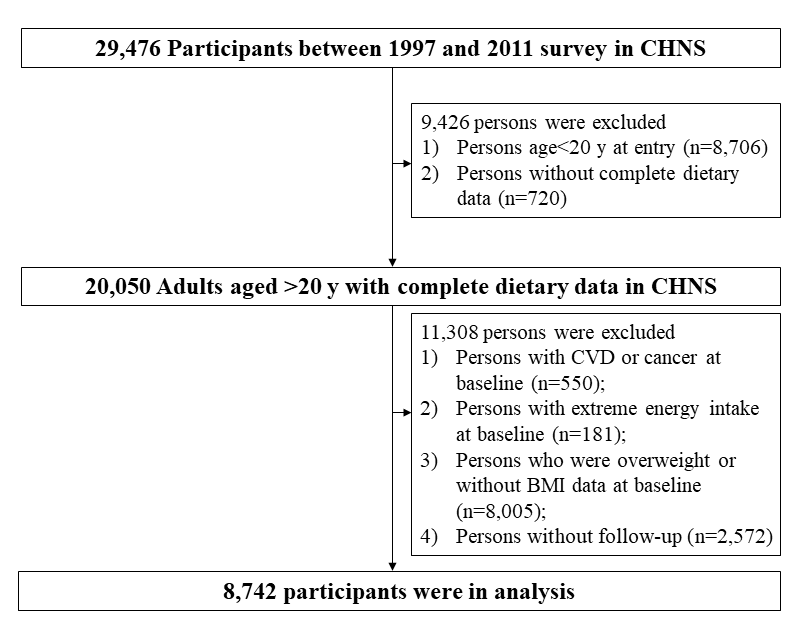


# Supplementary Figure. 1. Flow chart of study participants for analyses


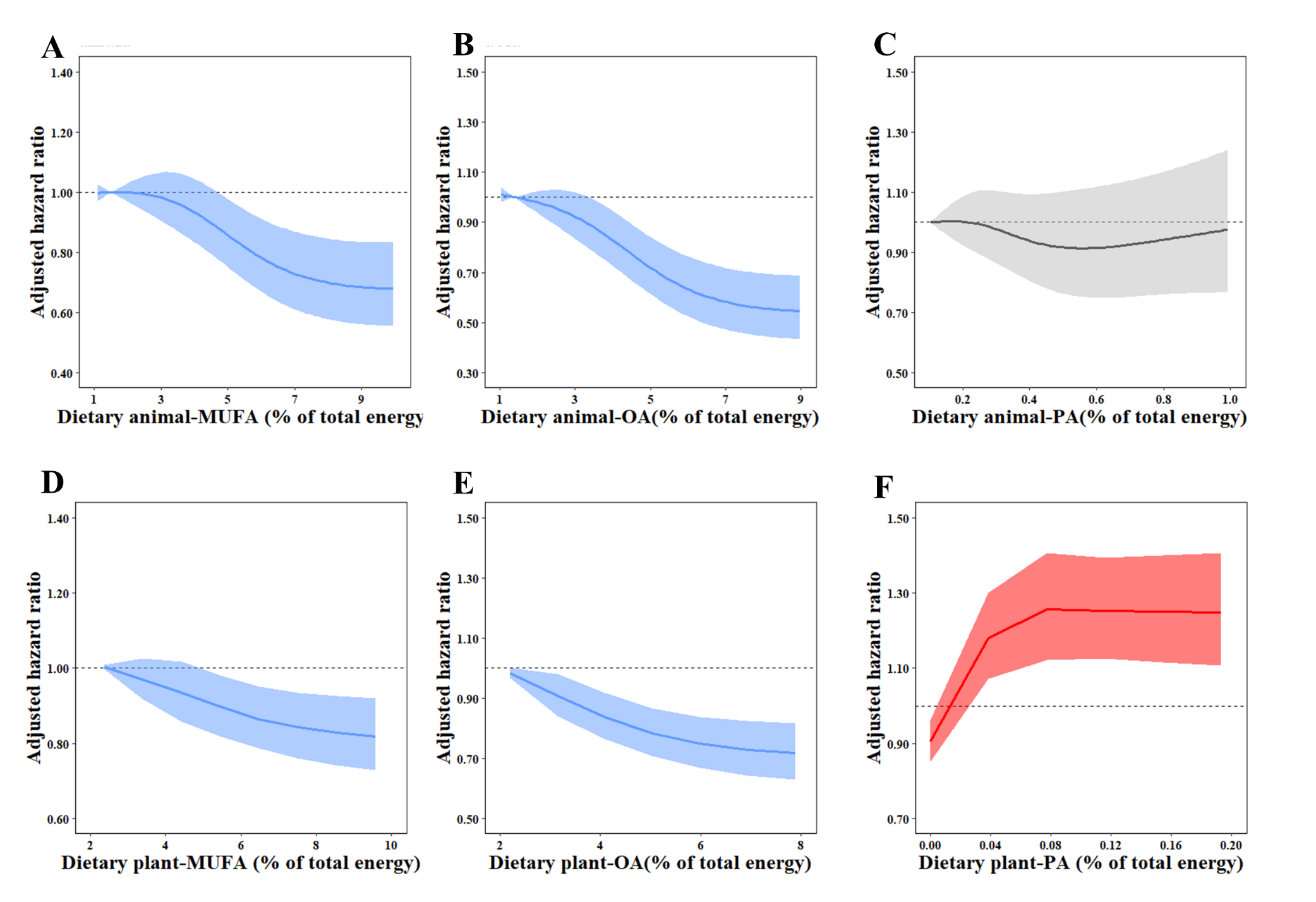


# Supplementary Figure. 2. Dose-response relationships between dietary MUFA intake and overweight/obesity risk. HRs for overweight/obesity associated with dietary animal-MUFAs (A), animal-OA (B), animal-PA (C), plant-MUFAs (D), plant-OA (E) and plant-PA (F) were estimated by restricted cubic-spline regression adjusted for age and sex, marital status, BMI, household income, urbanization index, nationality, education, physical activity, smoking, alcohol drinking status, history of hypertension and diabetes, total energy intake, percentages of energy intake from protein, SFAs and remaining fatty acids where appropriate. MUFA, monounsaturated fatty acid; OA, oleic acid; PA, palmitoleic acid.


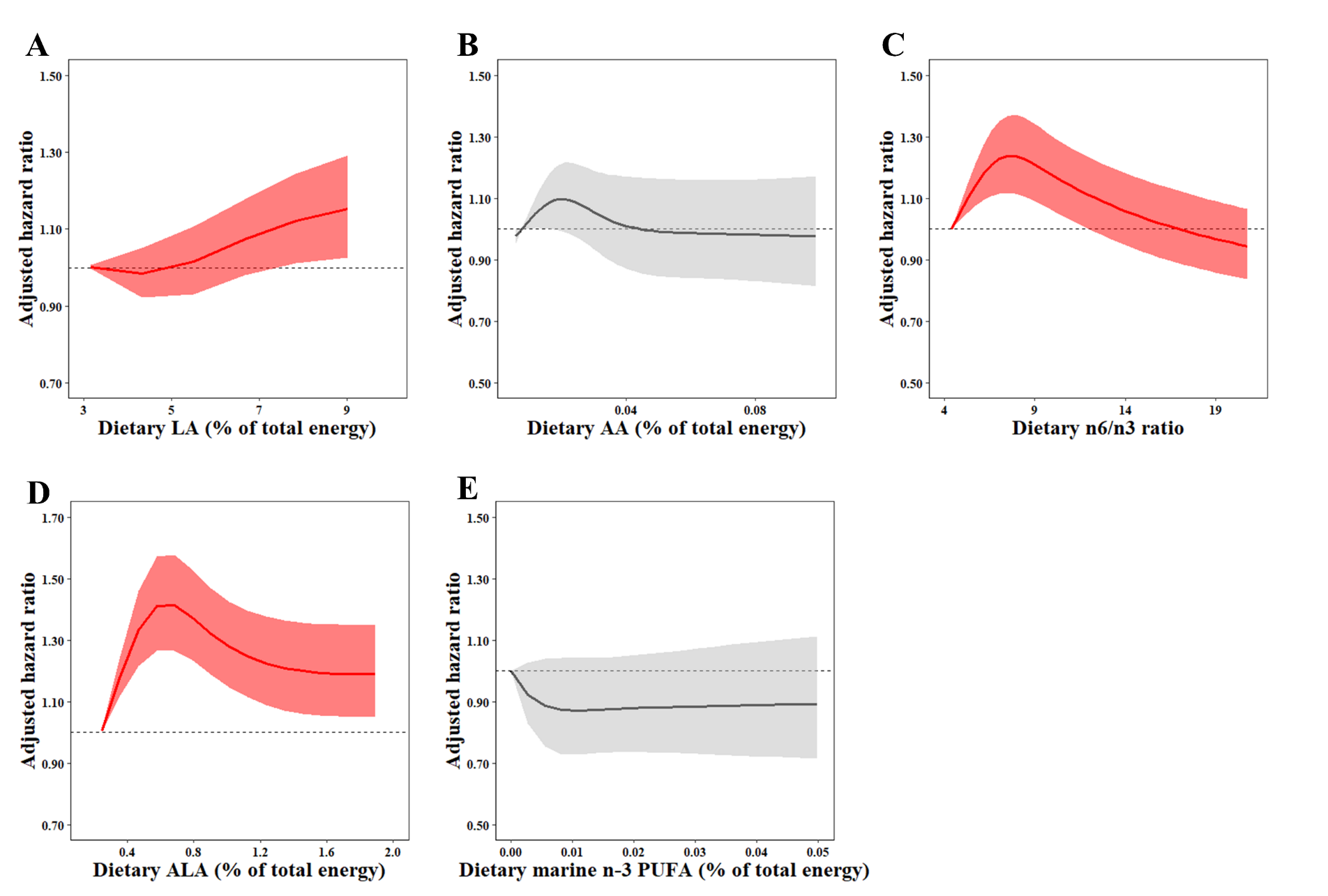


# Supplementary Figure. 3. Dose-response relationships between dietary PUFA intake and overweight/obesity risk. HRs for overweight/obesity associated with dietary LA (A), AA (B), n-6/n-3 PUFA ratio (C), ALA (D) and marine n-3 PUFAs (E) were estimated by restricted cubic-spline regression adjusted for age and sex, marital status, BMI, household income, urbanization index, nationality, education, physical activity, smoking, alcohol drinking status, history of hypertension and diabetes, total energy intake, percentages of energy intake from protein, SFA and remaining fatty acids where appropriate. LA, linoleic acid; AA, arachidonic acid; ALA, α-linolenic acid; PUFA, polyunsaturated fatty acid.

# Supplementary Table 1. Multivariable HRs (95% CIs) of overweight/obesity incidence according to the dietary intake of OA/PA from plants and animals

|  | **Quartiles of dietary fatty acids (% of total energy)** | | | | ***P-*trend^*^** |
| --- | --- | --- | --- | --- | --- |
|  | **Q1** | **Q2** | **Q3** | **Q4** |  |
| **P-OA** |  |  |  |  |  |
| Range | ≤ 2.7 | 2.7-4.0 | 4.0-5.5 | ≥ 5.5 |  |
| Median | 2.0 | 3.3 | 4.6 | 7.0 |  |
| Cases/ person-years | 617/8,740 | 734/15,302 | 730/19,674 | 672/19,665 |  |
| Model |  |  |  |  |  |
| 1.Overweight/obesity ~ Age, sex | 1.00 (ref.) | 0.96 (0.86-1.07) | 0.86 (0.77-0.96) | 0.89 (0.79-0.99) | 0.021 |
| 2. Overweight/obesity ~ Age, sex, BMI, marital status, household income, urbanization index, nationality, education, physical activity, smoking, alcohol drinking status, history of hypertension and diabetes | 1.00 (ref.) | 0.98 (0.88-1.09) | 0.88 (0.79-0.98) | 0.87 (0.77-0.97) | 0.005 |
| 3. Overweight/obesity ~ Age, sex, BMI, marital status, household income, urbanization index, nationality, education, physical activity, smoking, alcohol drinking status, history of hypertension and diabetes, total energy intake, percentages of energy intake from protein, SFAs, PUFAs, A-OA, PA | 1.00 (ref.) | 0.90 (0.81-1.01) | 0.78 (0.69-0.88) | 0.73 (0.64-0.83) | <0.001 |
| **A-OA** |  |  |  |  |  |
| Range | ≤ 2.7 | 2.7-4.5 | 4.5-6.6 | ≥ 6.6 |  |
| Median | 1.4 | 3.7 | 5.5 | 8.3 |  |
| Cases/ person-years | 716/19,665 | 755/19,674 | 702/15,302 | 580/13,110 |  |
| Model |  |  |  |  |  |
| 1.Overweight/obesity ~ Age, sex | 1.00 (ref.) | 1.11 (1.00-1.23) | 1.06 (0.95-1.17) | 1.02 (0.91-1.14) | 0.872 |
| 2. Overweight/obesity ~ Age, sex, BMI, marital status, household income, urbanization index, nationality, education, physical activity, smoking, alcohol drinking status, history of hypertension and diabetes | 1.00 (ref.) | 1.02 (0.91-1.13) | 0.94 (0.84-1.05) | 0.85 (0.75-0.96) | 0.004 |
| 3. Overweight/obesity ~ Age, sex, BMI, marital status, household income, urbanization index, nationality, education, physical activity, smoking, alcohol drinking status, history of hypertension and diabetes, total energy intake, percentages of energy intake from protein, SFAs, PUFAs, P-OA, PA | 1.00 (ref.) | 0.95 (0.82-1.09) | 0.78 (0.65-0.93) | 0.68 (0.55-0.84) | < 0.001 |
| **P-PA** |  |  |  |  |  |
| Range | ≤ 0.02 | 0.02-0.05 | 0.05-0.12 | ≥ 0.12 |  |
| Median | 0.01 | 0.03 | 0.08 | 0.19 |  |
| Cases/ person-years | 532/13,110 | 694/15,302 | 740/15,302 | 787/19,665 |  |
| Model |  |  |  |  |  |
| 1.Overweight/obesity ~ Age, sex | 1.00 (ref.) | 1.26 (1.13-1.41) | 1.28 (1.15-1.44) | 1.29 (1.16-1.45) | 0.001 |
| 2. Overweight/obesity ~ Age, sex, BMI, marital status, household income, urbanization index, nationality, education, physical activity, smoking, alcohol drinking status, history of hypertension and diabetes | 1.00 (ref.) | 1.25 (1.12-1.40) | 1.30 (1.17-1.46) | 1.35 (1.21-1.51) | < 0.001 |
| 3. Overweight/obesity ~ Age, sex, BMI, marital status, household income, urbanization index, nationality, education, physical activity, smoking, alcohol drinking status, history of hypertension and diabetes, total energy intake, percentages of energy intake from protein, SFAs, PUFAs, A-PA, OA | 1.00 (ref.) | 1.20 (1.06-1.35) | 1.23 (1.09-1.39) | 1.29 (1.14-1.47) | 0.002 |
| **A-PA** |  |  |  |  |  |
| Range | ≤ 0.2 | 0.2-0.3 | 0.3-0.5 | ≥ 0.5 |  |
| Median | 0.1 | 0.3 | 0.4 | 0.6 |  |
| Cases/ person-years | 726/19,665 | 745/19,674 | 706/15,302 | 576/10,925 |  |
| Model |  |  |  |  |  |
| 1.Overweight/obesity ~ Age, sex | 1.00 (ref.) | 1.04 (0.94-1.15) | 1.06 (0.95-1.17) | 1.05 (0.94-1.17) | 0.37 |
| 2. Overweight/obesity ~ Age, sex, BMI, marital status, household income, urbanization index, nationality, education, physical activity, smoking, alcohol drinking status, history of hypertension and diabetes | 1.00 (ref.) | 0.96 (0.87-1.07) | 0.91 (0.81-1.02) | 0.83 (0.73-0.94) | 0.002 |
| 3. Overweight/obesity ~ Age, sex, BMI, marital status, household income, urbanization index, nationality, education, physical activity, smoking, alcohol drinking status, history of hypertension and diabetes, total energy intake, percentages of energy intake from protein, SFAs, PUFAs, P-PA, OA | 1.00 (ref.) | 0.96 (0.84-1.09) | 0.90 (0.76-1.05) | 0.86 (0.71-1.05) | 0.132 |

OA, oleic acid; PA, palmitoleic acid; Q, quartile; HRs, hazard risks; CIs, confidence intervals.

**^*^** *P*-trend was assessed by calculating the median values in each quartile as continuous variables**.**

# Supplementary Table 2. Multivariable HRs (95% CIs) of overweight/obesity incidence according to PUFA subclass

|  | **Quartiles of dietary fatty acids (% of total energy)** | | | | ***P-*trend^*^** |
| --- | --- | --- | --- | --- | --- |
|  | **Q1** | **Q2** | **Q3** | **Q4** |  |
| **LA** |  |  |  |  |  |
| Range | ≤ 4.1 | 4.1-5.6 | 5.5-7.5 | ≥ 7.5 |  |
| Median | 3.2 | 4.8 | 6.4 | 9.5 |  |
| Cases/ person-years | 639/15,295 | 655/15,302 | 732/19,674 | 727/15,295 |  |
| Model |  |  |  |  |  |
| 1.Overweight/obesity ~ Age, sex | 1.00 (ref.) | 1.01 (0.90-1.12) | 1.12 (1.01-1.25) | 1.27 (1.14-1.41) | <0.001 |
| 2. Overweight/obesity ~ Age, sex, BMI, marital status, household income, urbanization index, nationality, education, physical activity, smoking, alcohol drinking status, history of hypertension and diabetes | 1.00 (ref.) | 0.95 (0.85-1.07) | 1.02 (0.92-1.14) | 1.13 (1.01-1.27) | 0.005 |
| 3. Overweight/obesity ~ Age, sex, BMI, marital status, household income, urbanization index, nationality, education, physical activity, smoking, alcohol drinking status, history of hypertension and diabetes, total energy intake, percentages of energy intake from protein, SFAs, MUFAs, AA, n-3 PUFAs | 1.00 (ref.) | 0.93 (0.83-1.05) | 1.01 (0.90-1.14) | 1.11 (0.98-1.26) | 0.02 |
| **AA** |  |  |  |  |  |
| Range | ≤ 0.01 | 0.01-0.02 | 0.02-0.04 | ≥ 0.04 |  |
| Median | 0.008 | 0.02 | 0.03 | 0.05 |  |
| Cases/ person-years | 683/19,665 | 727/19,674 | 735/15,302 | 608/13,110 |  |
| Model |  |  |  |  |  |
| 1.Overweight/obesity ~ Age, sex | 1.00 (ref.) | 1.11 (1.00-1.23) | 1.21 (1.09-1.34) | 1.18 (1.06-1.32) | 0.001 |
| 2. Overweight/obesity ~ Age, sex, BMI, marital status, household income, urbanization index, nationality, education, physical activity, smoking, alcohol drinking status, history of hypertension and diabetes | 1.00 (ref.) | 1.04 (0.94-1.16) | 1.08 (0.96-1.21) | 0.95 (0.84-1.08) | 0.435 |
| 3. Overweight/obesity ~ Age, sex, BMI, marital status, household income, urbanization index, nationality, education, physical activity, smoking, alcohol drinking status, history of hypertension and diabetes, total energy intake, percentages of energy intake from protein, SFAs, MUFAs, LA, n-3 PUFAs | 1.00 (ref.) | 1.06 (0.94-1.19) | 1.08 (0.94-1.24) | 0.96 (0.82-1.13) | 0.515 |
| **ALA** |  |  |  |  |  |
| Range | ≤ 0.37 | 0.37-0.68 | 0.67-1.06 | ≥ 1.06 |  |
| Median | 0.24 | 0.52 | 0.84 | 1.37 |  |
| Cases/ person-years | 590/19,665 | 705/13,116 | 744/15,302 | 714/19,665 |  |
| Model |  |  |  |  |  |
| 1.Overweight/obesity ~ Age, sex | 1.00 (ref.) | 1.50 (1.34-1.67) | 1.41 (1.27-1.57) | 1.34 (1.20-1.49) | <0.001 |
| 2. Overweight/obesity ~ Age, sex, BMI, marital status, household income, urbanization index, nationality, education, physical activity, smoking, alcohol drinking status, history of hypertension and diabetes | 1.00 (ref.) | 1.41 (1.26-1.58) | 1.34 (1.20-1.49) | 1.26 (1.13-1.41) | 0.006 |
| 3. Overweight/obesity ~ Age, sex, BMI, marital status, household income, urbanization index, nationality, education, physical activity, smoking, alcohol drinking status, history of hypertension and diabetes, total energy intake, percentages of energy intake from protein, SFAs, MUFAs, DPA, DHA, EPA, n-6 PUFAs | 1.00 (ref.) | 1.38 (1.23-1.55) | 1.32 (1.17-1.48) | 1.22 (1.07-1.39) | 0.039 |
| **marine n-3 PUFAs** |  |  |  |  |  |
| Range | 0 | 0-0.003 | 0.002-0.016 | ≥ 0.016 |  |
| Median | 0 | 0.001 | 0.008 | 0.035 |  |
| Cases/ person-years | 929/19,500 | 372/10,089 | 755/19,674 | 697/15,295 |  |
| Model |  |  |  |  |  |
| 1.Overweight/obesity ~ Age, sex | 1.00 (ref.) | 0.89 (0.79-1.01) | 0.97 (0.88-1.07) | 1.09 (0.99-1.21) | 0.039 |
| 2. Overweight/obesity ~ Age, sex, BMI, marital status, household income, urbanization index, nationality, education, physical activity, smoking, alcohol drinking status, history of hypertension and diabetes | 1.00 (ref.) | 0.89 (0.78-1.00) | 0.91 (0.83-1.01) | 0.96 (0.86-1.06) | 0.971 |
| 3. Overweight/obesity ~ Age, sex, BMI, marital status, household income, urbanization index, nationality, education, physical activity, smoking, alcohol drinking status, history of hypertension and diabetes, total energy intake, percentages of energy intake from protein, SFAs, MUFAs, DPA, ALA, n-6 PUFAs | 1.00 (ref.) | 0.93 (0.79-1.08) | 0.88 (0.74-1.04) | 0.83 (0.68-1.02) | 0.176 |

LA, linoleic acid; AA, arachidonic acid; ALA, α-linolenic acid; PUFA, polyunsaturated fatty acid; Q, quartile; HRs, hazard risks; CIs, confidence intervals; EPA, eicosapentaenoic acid; DHA, docosahexaenoic acid.; DPA, docosapentaenoic acid.

**^*^** *P*-trend was assessed by calculating the median values in each quartile as continuous variables**.**

# Supplementary Table 3. Subgroup analyses for multivariable HRs (95% CIs) of overweight/obesity incidence according to specific unsaturated FA intake

|  | **Quartiles of MUFA intake (% of total energy)** | | | |  |  | **Quartiles of PUFA intake (% of total energy)** | | | |  |  |
| --- | --- | --- | --- | --- | --- | --- | --- | --- | --- | --- | --- | --- |
| **Covariates** | **Q1** | **Q2** | **Q3** | **Q4** | ***P-*trend^*^** | ***P* for interaction** | **Q1** | **Q2** | **Q3** | **Q4** | ***P-*trend^*^** | ***P* for interaction** |
| **Age** |  |  |  |  |  |  |  |  |  |  |  |  |
| <50 yr | 1 | 1.03 (0.89-1.20) | 0.98 (0.82-1.18) | 0.94 (0.75-1.16) | 0.456 | 0.719 | 1 | 1.02 (0.90-1.17) | 1.15 (1.01-1.31) | 1.19 (1.04-1.37) | 0.003 | 0.226 |
| ≥50 yr | 1 | 0.99 (0.97-1.02) | 0.95 (0.91-0.98) | 0.59 (0.42-0.83) | 0.002 |  | 1 | 1.14 (0.90-1.44) | 1.23 (0.97-1.55) | 1.35 (1.07-1.71) | 0.011 |  |
| **Sex** |  |  |  |  |  |  |  |  |  |  |  |  |
| Men | 1 | 0.89 (0.74-1.07) | 0.91 (0.73-1.14) | 0.80 (0.62-1.05) | 0.133 | 0.041 | 1 | 0.92 (0.78-1.07) | 1.11 (0.95-1.30) | 1.18 (1.00-1.39) | 0.009 | 0.186 |
| Women | 1 | 0.99 (0.82-1.19) | 0.80 (0.64-0.99) | 0.78 (0.61-1.00) | 0.025 |  | 1 | 1.19 (1.01-1.40) | 1.24 (1.05-1.47) | 1.31 (1.11-1.54) | 0.003 |  |
| **Smoking status** | |  |  |  |  |  |  |  |  |  |  |  |
| Never or former | 1 | 0.98 (0.83-1.15) | 0.81 (0.67-0.98) | 0.79 (0.63-0.98) | 0.016 | 0.421 | 1 | 1.07 (0.93-1.23) | 1.21 (1.05-1.39) | 1.20 (1.04-1.38) | 0.007 | 0.080 |
| Current | 1 | 0.89 (0.71-1.11) | 0.97 (0.74-1.27) | 0.84 (0.60-1.16) | 0.363 |  | 1 | 0.99 (0.82-1.21) | 1.08 (0.88-1.31) | 1.30 (1.06-1.60) | 0.006 |  |
| **Alcohol drinking** |  |  |  |  |  |  |  |  |  |  |  |  |
| Non-drinker | 1 | 0.95 (0.81-1.12) | 0.81 (0.67-0.99) | 0.71 (0.56-0.88) | 0.001 | <0.001 | 1 | 1.08 (0.93-1.25) | 1.17 (1.01-1.35) | 1.25 (1.08-1.44) | 0.002 | 0.177 |
| Drinker | 1 | 0.95 (0.77-1.18) | 0.94 (0.73-1.23) | 0.99 (0.73-1.34) | 0.969 |  | 1 | 0.99 (0.82-1.19) | 1.19 (0.99-1.43) | 1.21 (1.00-1.48) | 0.015 |  |
| **Physical activity** | |  |  |  |  |  |  |  |  |  |  |  |
| Low | 1 | 0.92 (0.71-1.20) | 0.82 (0.61-1.09) | 0.70 (0.51-0.95) | 0.008 | 0.040 | 1 | 0.96 (0.77-1.19) | 1.07 (0.87-1.33) | 1.10 (0.89-1.36) | 0.148 | 0.143 |
| Moderate to vigorous | 1 | 0.98 (0.84-1.15) | 0.89 (0.73-1.09) | 0.95 (0.74-1.21) | 0.528 |  | 1 | 1.05 (0.91-1.21) | 1.19 (1.03-1.38) | 1.21 (1.04-1.41) | 0.006 |  |
| **Education** | |  |  |  |  |  |  |  |  |  |  |  |
| Below great high school | 1 | 0.94 (0.82-1.08) | 0.89 (0.75-1.04) | 0.86 (0.71-1.04) | 0.110 | 0.012 | 1 | 1.08 (0.96-1.22) | 1.15 (1.02-1.30) | 1.26 (1.12-1.43) | <0.001 | 0.150 |
| Great high school and above | 1 | 0.96 (0.55-1.69) | 0.69 (0.37-1.30) | 0.48 (0.24-0.95) | 0.002 |  | 1 | 0.63 (0.41-0.97) | 1.09 (0.73-1.63) | 0.88 (0.59-1.31) | 0.655 |  |
| **household income** | | |  |  |  |  |  |  |  |  |  |  |
| < median | 1 | 1.03 (0.87-1.21) | 0.89 (0.73-1.10) | 0.81 (0.63-1.05) | 0.098 | 0.901 | 1 | 1.06 (0.95-1.19) | 1.16 (1.03-1.30) | 1.21 (1.08-1.36) | <0.001 | 0.007 |
| > median | 1 | 0.88 (0.71-1.09) | 0.82 (0.64-1.05) | 0.81 (0.61-1.06) | 0.158 |  | 1 | 0.90 (0.65-1.26) | 1.11 (0.81-1.52) | 1.16 (0.83-1.63) | 0.442 |  |
| **history of hypertension** | | |  |  |  |  |  |  |  |  |  |  |
| No | 1 | 0.97 (0.84-1.11) | 0.87 (0.74-1.02) | 0.77 (0.64-0.93) | 0.004 | 0.662 | 1 | 1.07 (0.95-1.21) | 1.20 (1.07-1.36) | 1.28 (1.13-1.45) | <0.001 | 0.467 |
| Yes | 1 | 0.81 (0.53-1.24) | 0.76 (0.45-1.26) | 1.12 (0.64-1.98) | 0.537 |  | 1 | 0.83 (0.58-1.20) | 0.88 (0.60-1.28) | 0.89 (0.59-1.35) | 0.742 |  |
| **history of diabetes** | | |  |  |  |  |  |  |  |  |  |  |
| No | 1 | 0.95 (0.83-1.08) | 0.85 (0.72-0.99) | 0.81 (0.68-0.98) | 0.019 | 0.287 | 1 | 1.03 (0.92-1.16) | 1.16 (1.04-1.30) | 1.23 (1.10-1.38) | <0.001 | 0.674 |
| Yes | 1 | 1.18 (0.34-4.13) | 1.05 (0.23-4.74) | 0.30 (0.05-1.70) | 0.090 |  | 1 | 4.19 (1.01-17.43) | 2.46 (0.48-12.70) | 3.60 (0.77-16.82) | 0.403 |  |

MUFA, monounsaturated fatty acid; PUFA, polyunsaturated fatty acid; Q, quartile; HRs, hazard risks; CIs, confidence intervals.

**^*^** *P*-trend was assessed by calculating the median values in each quartile as continuous variables**.****Supplementary Table 4.** Sensitivity analyses for multivariable HRs (95% CIs) of overweight/obesity incidence according to dietary MUFA intake

|  | **Quartiles of plasma fatty acids (% of total fatty acids)** | | |  |  |
| --- | --- | --- | --- | --- | --- |
|  | Q1 | Q2 | Q3 | Q4 | *P-*trend**^*^** |
| **Excluding participants with extreme BMI (<18.5)** | |  |  |  |  |
| MUFAs | 1 | 0.96 (0.84-1.09) | 0.85 (0.73-1.00) | 0.81 (0.67-0.97) | 0.015 |
| OA | 1 | 0.93 (0.82-1.06) | 0.83 (0.71-0.97) | 0.67 (0.56-0.80) | <0.001 |
| PA | 1 | 1.05 (0.93-1.19) | 1.15 (0.99-1.34) | 1.15 (0.96-1.38) | 0.140 |
| A-MUFAs | 1 | 0.99 (0.86-1.13) | 0.85 (0.72-1.00) | 0.77 (0.64-0.94) | 0.005 |
| P-MUFAs | 1 | 0.93 (0.83-1.04) | 0.94 (0.84-1.05) | 0.83 (0.73-0.94) | 0.004 |
| A-PA | 1 | 0.97 (0.85-1.10) | 0.91 (0.77-1.07) | 0.87 (0.72-1.06) | 0.004 |
| P-PA | 1 | 1.20 (1.07-1.36) | 1.22 (1.08-1.38) | 1.28 (1.13-1.46) | 0.005 |
| A-OA | 1 | 0.95 (0.83-1.09) | 0.78 (0.65-0.93) | 0.67 (0.54-0.83) | <0.001 |
| P-OA | 1 | 0.89 (0.80-1.00) | 0.79 (0.70-0.89) | 0.72 (0.63-0.82) | <0.001 |
| **Further adjusting for cholesterol intake** | | | |  |  |
| MUFAs | 1 | 0.94 (0.83-1.08) | 0.84 (0.72-0.98) | 0.79 (0.66-0.94) | 0.007 |
| OA | 1 | 0.93 (0.82-1.05) | 0.82 (0.71-0.96) | 0.66 (0.55-0.79) | <0.001 |
| PA | 1 | 1.03 (0.91-1.17) | 1.13 (0.97-1.32) | 1.11 (0.93-1.34) | 0.244 |
| A-MUFAs | 1 | 0.96 (0.84-1.10) | 0.81 (0.69-0.96) | 0.74 (0.61-0.90) | 0.001 |
| P-MUFAs | 1 | 0.93 (0.83-1.04) | 0.94 (0.83-1.05) | 0.83 (0.73-0.93) | 0.003 |
| A-PA | 1 | 0.92 (0.80-1.05) | 0.84 (0.71-0.99) | 0.80 (0.66-0.98) | 0.038 |
| P-PA | 1 | 1.20 (1.06-1.35) | 1.24 (1.10-1.40) | 1.29 (1.13-1.46) | 0.003 |
| A-OA | 1 | 0.94 (0.81-1.08) | 0.77 (0.65-0.92) | 0.67 (0.54-0.83) | <0.001 |
| P-OA | 1 | 0.91 (0.81-1.01) | 0.78 (0.70-0.88) | 0.73 (0.64-0.83) | <0.001 |
| **Further adjusting for AHEI** | | |  |  |  |
| MUFAs | 1 | 0.96 (0.84-1.10) | 0.88 (0.75-1.02) | 0.82 (0.68-0.98) | 0.024 |
| OA | 1 | 0.94 (0.83-1.07) | 0.84 (0.72-0.98) | 0.68 (0.57-0.82) | <0.001 |
| PA | 1 | 1.05 (0.93-1.19) | 1.14 (0.98-1.33) | 1.10 (0.92-1.33) | 0.180 |
| A-MUFAs | 1 | 1.00 (0.87-1.14) | 0.86 (0.73-1.01) | 0.79 (0.65-0.96) | 0.010 |
| P-MUFAs | 1 | 0.93 (0.83-1.04) | 0.95 (0.85-1.06) | 0.84 (0.74-0.95) | 0.008 |
| A-PA | 1 | 0.96 (0.84-1.09) | 0.90 (0.76-1.05) | 0.86 (0.70-1.04) | 0.107 |
| P-PA | 1 | 1.20 (1.07-1.35) | 1.24 (1.10-1.39) | 1.29 (1.13-1.46) | 0.003 |
| A-OA | 1 | 0.95 (0.83-1.10) | 0.80 (0.67-0.95) | 0.69 (0.56-0.86) | <0.001 |
| P-OA | 1 | 0.91 (0.81-1.02) | 0.79 (0.70-0.89) | 0.74 (0.65-0.85) | <0.001 |
| **Further adjusting for occupation** | | |  |  |  |
| MUFAs | 1 | 0.96 (0.84-1.09) | 0.86 (0.74-1.01) | 0.79 (0.66-0.95) | 0.008 |
| OA | 1 | 0.94 (0.83-1.07) | 0.84 (0.72-0.97) | 0.67 (0.56-0.80) | <0.001 |
| PA | 1 | 1.06 (0.93-1.19) | 1.17 (1.01-1.35) | 1.15 (0.96-1.38) | 0.121 |
| A-MUFAs | 1 | 0.99 (0.87-1.13) | 0.85 (0.72-1.00) | 0.78 (0.64-0.95) | 0.006 |
| P-MUFAs | 1 | 0.93 (0.83-1.04) | 0.94 (0.84-1.05) | 0.82 (0.73-0.93) | 0.002 |
| A-PA | 1 | 0.97 (0.85-1.10) | 0.90 (0.77-1.06) | 0.87 (0.72-1.06) | 0.164 |
| P-PA | 1 | 1.18 (1.05-1.33) | 1.22 (1.08-1.37) | 1.28 (1.13-1.45) | 0.003 |
| A-OA | 1 | 0.95 (0.83-1.09) | 0.78 (0.66-0.93) | 0.68 (0.55-0.85) | <0.001 |
| P-OA | 1 | 0.90 (0.81-1.01) | 0.79 (0.70-0.89) | 0.73 (0.64-0.83) | <0.001 |
| **Excluding participants with hypertension or diabetes at baseline** | | |  |  |  |
| MUFAs | 1 | 0.97 (0.84-1.11) | 0.86 (0.73-1.01) | 0.79 (0.65-0.96) | 0.009 |
| OA | 1 | 0.92 (0.80-1.05) | 0.82 (0.69-0.96) | 0.65 (0.54-0.79) | <0.001 |
| PA | 1 | 1.05 (0.92-1.20) | 1.17 (1.00-1.37) | 1.14 (0.94-1.38) | 0.193 |
| A-MUFAs | 1 | 0.97 (0.84-1.11) | 0.80 (0.67-0.95) | 0.73 (0.59-0.89) | 0.001 |
| P-MUFAs | 1 | 0.92 (0.81-1.03) | 0.96 (0.85-1.08) | 0.82 (0.72-0.94) | 0.006 |
| A-PA | 1 | 0.96 (0.83-1.10) | 0.88 (0.74-1.04) | 0.85 (0.69-1.04) | 0.106 |
| P-PA | 1 | 1.24 (1.09-1.41) | 1.28 (1.13-1.46) | 1.33 (1.16-1.52) | 0.003 |
| A-OA | 1 | 0.93 (0.80-1.08) | 0.74 (0.61-0.89) | 0.64 (0.51-0.80) | <0.001 |
| P-OA | 1 | 0.89 (0.79-1.00) | 0.77 (0.68-0.87) | 0.72 (0.63-0.83) | <0.001 |

HRs, hazard risks; CIs, confidence intervals; Q, quartile; MUFA, monounsaturated fatty acid; OA, oleic acid; PA, palmitoleic acid. AHEI, Alternative Healthy Eating Index.

**^*^** *P*-trend was assessed by calculating the median values in each quartile as continuous variables**.**

#

# Supplementary Table 5. Sensitivity analyses for multivariable HRs (95% CIs) of overweight/obesity incidence according to dietary PUFA intake

|  | **Quartiles of plasma fatty acids (% of total fatty acids)** | | |  |  |
| --- | --- | --- | --- | --- | --- |
|  | Q1 | Q2 | Q3 | Q4 | *P-*trend**^*^** |
| **Excluding participants with extreme BMI (<18.5)** | | | |  |  |
| PUFAs | 1 | 1.06 (0.95-1.19) | 1.19 (1.06-1.34) | 1.25 (1.11-1.41) | <0.001 |
| n-3 PUFAs | 1 | 1.38 (1.23-1.55) | 1.29 (1.15-1.45) | 1.25 (1.10-1.43) | 0.014 |
| marine n-3 PUFAs | 1 | 0.91 (0.78-1.07) | 0.87 (0.73-1.03) | 0.83 (0.68-1.01) | 0.202 |
| ALA | 1 | 1.36 (1.21-1.53) | 1.31 (1.17-1.47) | 1.22 (1.07-1.39) | 0.036 |
| n-6 PUFAs | 1 | 0.97 (0.86-1.09) | 1.03 (0.92-1.16) | 1.14 (1.00-1.29) | 0.015 |
| LA | 1 | 0.96 (0.85-1.08) | 1.03 (0.91-1.16) | 1.12 (0.99-1.28) | 0.020 |
| AA | 1 | 1.05 (0.93-1.19) | 1.08 (0.94-1.24) | 0.97 (0.82-1.13) | 0.531 |
| n-6/n-3 PUFA ratio | 1 | 1.19 (1.06-1.33) | 1.21 (1.07-1.37) | 0.94 (0.83-1.06) | 0.003 |
| **Further adjusting for cholesterol intake** | | |  |  |  |
| PUFAs | 1 | 1.04 (0.92-1.16) | 1.15 (1.03-1.29) | 1.21 (1.08-1.36) | <0.001 |
| n-3 PUFAs | 1 | 1.39 (1.24-1.56) | 1.29 (1.15-1.44) | 1.24 (1.09-1.41) | 0.026 |
| marine n-3 PUFAs | 1 | 0.91 (0.78-1.06) | 0.86 (0.73-1.02) | 0.82 (0.67-1.00) | 0.170 |
| ALA | 1 | 1.38 (1.23-1.54) | 1.30 (1.16-1.46) | 1.21 (1.06-1.38) | 0.054 |
| n-6 PUFAs | 1 | 0.93 (0.83-1.05) | 1.00 (0.89-1.12) | 1.11 (0.98-1.26) | 0.025 |
| LA | 1 | 0.93 (0.83-1.04) | 1.00 (0.89-1.12) | 1.10 (0.97-1.25) | 0.035 |
| AA | 1 | 1.00 (0.88-1.13) | 1.01 (0.87-1.17) | 0.89 (0.75-1.06) | 0.126 |
| n-6/n-3 PUFA ratio | 1 | 1.20 (1.07-1.34) | 1.22 (1.08-1.37) | 0.94 (0.84-1.06) | 0.003 |
| **Further adjusting for AHEI** | |  |  |  |  |
| PUFAs | 1 | 1.04 (0.93-1.17) | 1.14 (1.01-1.29) | 1.18 (1.04-1.34) | 0.006 |
| n-3 PUFAs | 1 | 1.38 (1.23-1.55) | 1.29 (1.15-1.44) | 1.23 (1.08-1.40) | 0.034 |
| marine n-3 PUFAs | 1 | 0.93 (0.79-1.08) | 0.88 (0.74-1.04) | 0.82 (0.67-1.01) | 0.127 |
| ALA | 1 | 1.37 (1.22-1.54) | 1.30 (1.16-1.46) | 1.21 (1.06-1.38) | 0.056 |
| n-6 PUFAs | 1 | 0.94 (0.84-1.05) | 1.00 (0.89-1.13) | 1.10 (0.96-1.25) | 0.056 |
| LA | 1 | 0.93 (0.83-1.04) | 1.00 (0.89-1.12) | 1.08 (0.95-1.23) | 0.081 |
| AA | 1 | 1.06 (0.94-1.19) | 1.09 (0.95-1.25) | 0.97 (0.82-1.13) | 0.516 |
| n-6/n-3 PUFA ratio | 1 | 1.17 (1.05-1.32) | 1.19 (1.06-1.34) | 0.94 (0.84-1.06) | 0.007 |
| **Further adjusting for occupation** | | |  |  |  |
| PUFAs | 1 | 1.05 (0.94-1.18) | 1.18 (1.05-1.32) | 1.24 (1.10-1.39) | <0.001 |
| n-3 PUFAs | 1 | 1.38 (1.23-1.55) | 1.28 (1.14-1.44) | 1.22 (1.08-1.39) | 0.036 |
| marine n-3 PUFAs | 1 | 0.94 (0.80-1.09) | 0.88 (0.74-1.05) | 0.84 (0.69-1.03) | 0.197 |
| ALA | 1 | 1.37 (1.22-1.53) | 1.30 (1.16-1.46) | 1.20 (1.05-1.37) | 0.070 |
| n-6 PUFAs | 1 | 0.95 (0.84-1.06) | 1.02 (0.91-1.15) | 1.14 (1.00-1.29) | 0.009 |
| LA | 1 | 0.94 (0.84-1.05) | 1.02 (0.91-1.14) | 1.12 (0.99-1.27) | 0.014 |
| AA | 1 | 1.05 (0.94-1.19) | 1.09 (0.95-1.24) | 0.97 (0.83-1.14) | 0.595 |
| n-6/n-3 PUFA ratio | 1 | 1.20 (1.07-1.35) | 1.22 (1.09-1.38) | 0.96 (0.85-1.08) | 0.007 |
| **Excluding participants with hypertension or diabetes at baseline** | | | |  |  |
| PUFAs | 1 | 1.06 (0.94-1.20) | 1.20 (1.06-1.35) | 1.27 (1.13-1.44) | <0.001 |
| n-3 PUFAs | 1 | 1.38 (1.22-1.56) | 1.34 (1.19-1.51) | 1.25 (1.09-1.43) | 0.012 |
| marine n-3 PUFAs | 1 | 0.91 (0.77-1.08) | 0.84 (0.70-1.01) | 0.79 (0.63-0.98) | 0.111 |
| ALA | 1 | 1.36 (1.20-1.54) | 1.34 (1.19-1.52) | 1.23 (1.07-1.42) | 0.020 |
| n-6 PUFAs | 1 | 0.94 (0.83-1.06) | 1.05 (0.93-1.19) | 1.11 (0.97-1.26) | 0.035 |
| LA | 1 | 0.93 (0.82-1.05) | 1.05 (0.93-1.19) | 1.09 (0.96-1.25) | 0.044 |
| AA | 1 | 1.04 (0.92-1.18) | 1.03 (0.89-1.19) | 0.94 (0.79-1.11) | 0.312 |
| n-6/n-3 PUFA ratio | 1 | 1.20 (1.06-1.35) | 1.23 (1.08-1.40) | 0.93 (0.82-1.06) | 0.002 |

HRs, hazard risks; CIs, confidence intervals; Q, quartile; LA, linoleic acid; AA, arachidonic acid; ALA, α-linolenic acid; PUFA, polyunsaturated fatty acid; AHEI, Alternative Healthy Eating Index.

**^*^** *P*-trend was assessed by calculating the median values in each quartile as continuous variables**.**
